# Dentistry and General Medicine

**Published:** 1895-05

**Authors:** George H. Chance

**Affiliations:** Portland, Oregon.


ARTICLE II.

### DENTISTRY AND GENERAL MEDICINE.

GEORGE H. CHANCE, D. D. S., OF PORTLAND, OREGON.

However much American dentists may differ concerning the practice of dentistry and its relation to general medicine, medical men, with few exceptions, believe dentistry is a mechanical art only, and that dentists are mechanical craftmen, and as such are not entitled to professional recognition from medical men. It will, therefore, be the purpose of this paper to show, if possible, that whatever may have been the dentistry of the past, the dentistry of the present is both a science and an art, as well as a liberal profession.

Modern dentistry is, in the broadest sense, both a science and an art. As a science, it deals with a full and an accurate knowledge of the dental organs and their functions, as well as with a knowledge of the causes leading to their pathological conditions whereby disease is engendered, and function either impaired or destroyed.

As an art, its votaries are instructed in and made acquainted with the various methods and means for combating dental and oral disease, that health may return and, wherever possible, function restored; so that it is quite safe to say that dentistry is science applied to dental and oral surgery. And it is quite as safe to say that medicine, when stripped of its scientific adjuncts, major and minor surgery, is largely a system of guessing at probabilities in disease, with a fearful lack of harmony existing among the guessers, divided as they are into belligerent camps, some large and "regular," and some large and "irregular," besides other small camps of various construction, with guessing schools attached to each camp, teaching different theories, and each claiming to teach the only true Simon-pure method of guessing and of practice. And these, forsooth, are they who assume to write and publish their guessing criticisms on the dentist and his methods; one of these from the large and "regular" camp gravely guesses that dentists are not sufficiently sterilized to prevent infection from syphilitic patients when they apply to the dentist for treatment of the oral cavity.

Then there is the brother from the "irregular" camp of guessers, and he guesses that dentists ought not, under any circumstances, use amalgam for filling cavities in defective teeth, because he guesses that the potency of the dynamic force of his fifteenth dilution of medicinal dynamo will be destroyed by coming in contact with the wandering mercurial spooks, which he guesses may inadvertently escape from between the meshes of the amalgam plugs. But surely we are not as ignorant as some of these guessers take us to be, and really do know more about some things than they give us credit for.

Dentistry has discovered the cause, and is daily successfully treating 90 per cent. of all the cases of so-called "facial neuralgia," whose victims apply to the dentist for relief. It has also vastly improved on the methods formerly in vogue by the medical profession for the surgical treatment of tumors of the oral cavity, as well as fractures of the jaws, simple or compound. It has aided and abetted the aurist and oculist in their efforts to restore function. It was a dentist who first thought out and who first put to practical use that boon of boons, anæsthesia, and who gave his thoughts and his experience to the medical profession and to the world without fee or reward. What has here been stated as standing to the credit of dentistry is but a tithe of what it has accomplished, and is destined to yet accomplish in the interest of suffering humanity, in spite of adverse medical criticism.

The "medicine man," whose office was in former ages merged with that of the priest and prophet, and who also was thought by many to be gifted with supernatural powers in the art of healing the sick, has from time immemorial exerted a seeming mysterious influence over the minds of the common people, sometimes for good, and sometimes for evil; and this seemingly strange influence exists in the minds of the laity to day; hence, the physician's opinion, when he is called to the sick-room, is deferred to in all cases almost without question; and to a certain extent this is right, for such confidence enables the conscientious, competent physician to treat the patient according to the exigency of the case, free from the ignorant interferences of anxious friends. Besides this, very few of the laity possess any genuine knowledge of the *mysteries of medicine*, so that there are obvious reasons for this sometimes "blind faith" in the chosen physician, no matter from what school he may come.

Such things show us how very differently the physician and the dentist are held in public estimation; the services of the former being sought mainly for his supposed skill in the mysteries of medicine, while the latter is employed not so much for his technical knowledge, which, of course, he must possess, as for the special labor he is expected to perform; so that to succeed in our calling it is not necessary that we should wear the badge of medical servitude, for dentistry is the product of a higher and more advanced form of civilization—a scientific, independent profession, developed by master minds of a more recent generation, and not a *medical legacy* left us from a former period, when the medicine man was the only source to whom suffering humanity was taught to look for physical relief, and whose application was in vain when the malady was of dental origin.

Dentistry, like general medicine, is here to stay; both are needed, and both are without doubt intended as instruments in the hands of the properly educated for the relief of physical suffering; and so far as scientifically educated, limited human foresight can go, are also intended to be used for the *prevention of disease*; but such desired results can be reached in certain cases only in proportion as physician and dentist are willing to consult and co-operate with each other; and to do this intelligently and successfully each must possess that kind of an education which will not only fit him for practice in his own field, but must, to a considerable extent, overlap other branches of the healing art. While it may not take in all details, neither does such an education imply that a dentist shall practice general medicine, or the physician dental surgery.

This is precisely the kind of education the dental students acquire in the dental colleges of to-day; three full years of study and attendance on three full courses of lectures being required before the student can be graduated. It will be noticed that the time required to be graduated in dentistry is the same as that required for graduation in general medicine. It will therefore appear to the observant mind, that if it takes three years to graduate a student in dentistry, and no more than three years to graduate a student in general medicine, that there must be something more to learn in dentistry than the average physician, with his present limited knowledge of the dental organs, is prepared to admit. The medical man should, if only for his own reputation, be better informed on the subject of the teeth than is the average physician of to-day, the teaching of which has been sadly neglected in the medical colleges. —*Items of Interest.*

---

ARTICLE III.

### THE HORACE WELLS PERMANENT MEMORIAL.

